# Towards Realistic 3D-Printed Phantoms for Aneurysm Clipping: Mechanical Characterisation of Basilar Arteries

**DOI:** 10.3390/bioengineering12111239

**Published:** 2025-11-12

**Authors:** Pavel Buchvald, Lukas Capek, Petra Hamrikova, Jiri Safka, Jiri Vitvar

**Affiliations:** 1 Department of Neurosurgery, Regional Hospital in Liberec, 46001 Liberec, Czech Republic; 2 Department of Clinical Biomechanics, Regional Hospital in Liberec, 46001 Liberec, Czech Republic; 3 Department of Technologies and Structures, Faculty of Textile Engineering, Technical University of Liberec, 46001 Liberec, Czech Republic; 4 Department of Forensic Pathology, Regional Hospital in Liberec, 46001 Liberec, Czech Republic; 5 Advanced Technologies and Innovation, Institute for Nanomaterials, 46001 Liberec, Czech Republic; 6 Department of Medical Biophysics, Faculty of Medicine in Hradec Kralove, 50038 Hradec Kralove, Czech Republic

**Keywords:** 3D printing, brain aneurysm, phantom, in vitro experiments

## Abstract

Cerebral aneurysm clipping remains a key surgical approach despite advancements in endovascular techniques. However, training for this procedure is complex due to the variable and fragile nature of aneurysmal tissues. This study evaluates the mechanical behaviour of human basilar arteries during clipping and compares them to 3D-printed models used for neurosurgical training. Mechanical tests were performed on ten cadaveric basilar arteries, distinguishing between healthy and plaque-affected segments. Plaque-affected regions required significantly higher clipping force (1.73 ± 0.22 N) compared to healthy segments (0.45 ± 0.19 N), confirming that atherosclerosis markedly increases arterial stiffness. Six 3D-printed phantom materials were evaluated; none accurately replicated the biomechanical response of real arteries. The Flex Anatomical material showed the highest stiffness (44.51 ± 0.98 N), while Silicone 40A was the most compliant (1.05 ± 0.12 N), yet both deviated substantially from biological performance. These findings underscore the current limitations of anatomical models that lack realistic biomechanical properties.

## 1. Introduction

Cerebral aneurysms are localised tissue expansion of blood vessels in the brain that can pose a significant risk of rupture, leading to subarachnoid haemorrhage and potentially severe neurological impairment or death [1,2]. Even though there are several approaches to treating it, surgical clipping remains a cornerstone in the management of these vascular lesions, providing a definitive method for preventing rupture by occluding the aneurysm [3,4]. However, the complexities associated with aneurysm anatomy, including variations in size, shape, and location, present considerable challenges for neurosurgeons [5,6]. Finite element method (FEM) simulations have been used to analyse the mechanical behaviour of cerebral aneurysms during surgical clipping. These simulation studies focused on stress and strain distribution in the aneurysm wall, deformation during clip application, and the interaction between the clip and vessel tissue [7,8,9]. The models enhance biomechanical understanding of clipping and support the refinement of surgical methods for safer and more effective outcomes. On the other hand, the FEM simulations are still not used in daily clinical practice due to the complexity of this approach.

**Figure 1:**
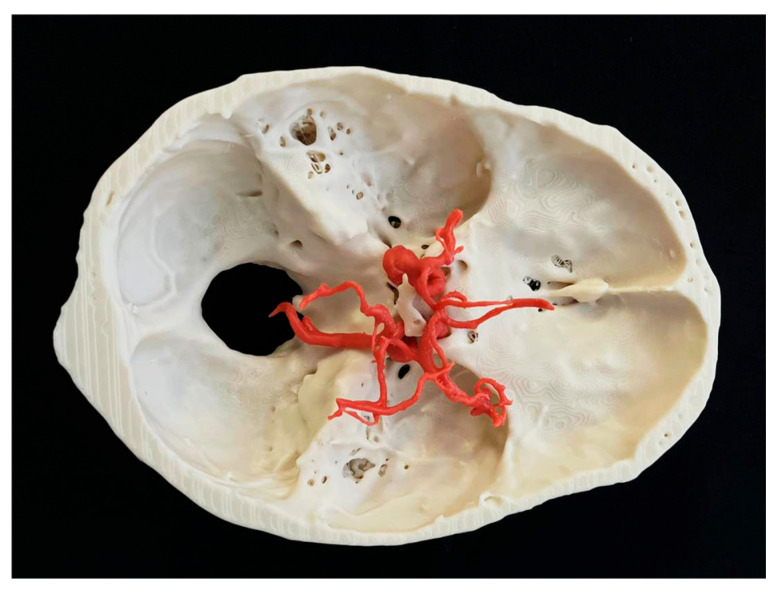
Three-dimensional patient-specific model of brain aneurysm with cranial base.

The intricacies of the surgical approach necessitate a high degree of precision and skill, underscoring the importance of comprehensive training in this critical area of neurosurgery. Traditionally, surgical training for aneurysm clipping has relied heavily on learning skills and supervised practice on patients [10,11,12]. While mentorship and hands-on experience are invaluable, the risks involved in operating on live patients can inhibit the learning process and limit opportunities for practice. One of the most promising advancements in surgical training is the use of 3D-printed models, as shown in Figure 1.

These models are created from patient-specific imaging data, such as computer tomography angiography (CTA) or magnetic resonance angiography (MRA) scans, and accurately replicate the anatomical features of individual aneurysms and their surrounding vascular structures [13,14,15,16,17,18]. By providing a tactile, realistic experience, 3D-printed models serve as effective training phantoms that allow neurosurgeons to practise complex techniques in a controlled environment. Trainees can refine their surgical skills, explore different approaches, and understand the nuances of aneurysm clipping without the associated risks of operating on live patients. Up to date, to our knowledge, there is no data-based study focusing on the mechanical behaviour of brain aneurysms during clipping. This article aims to explore the role of 3D-printed models as training phantoms in the context of brain aneurysm clipping and their ability to mimic a biological material.

## 2. Materials and Methods

**Figure 2:**
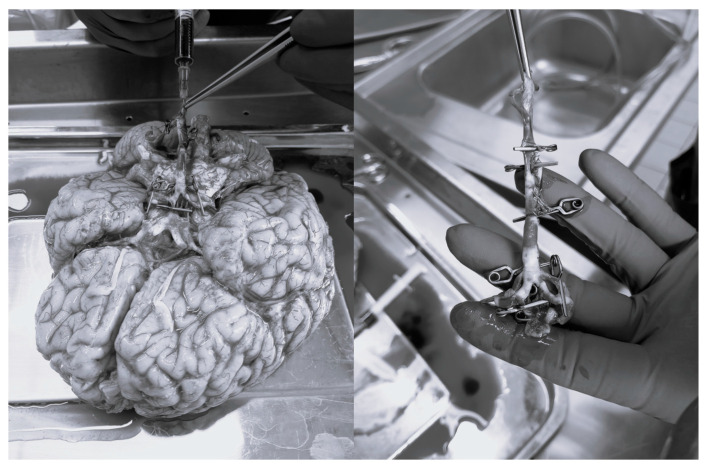
Preparation of basilar artery for experiments.

The study was conducted under ethical approval of the Local Ethics Committee (Regional Hospital Liberec, Liberec, Czech Republic) and in accordance with the Human Tissue Act 2004. The aim of the study is to find out the radial stiffness of the basilar artery and different 3D-printed materials. In a cadaveric study, fresh, intact, human-cadaveric Willis’s circle specimens were obtained from deceased donors with a mean age of 72 years (range 55–78). After dissection, the specimens were carefully cleaned, and cuts were made in the regions of the vertebral and basilar arteries by the lead author. The inlet and outlet entrances were closed using microclips (Yasargil, BBraun, Melsungen, Germany) and pressured via Betadine solution, as shown in Figure 2. The sclerotic artery areas were marked, and the diameter of the artery was measured. Group 1 comprised 10 specimens with sclerotic arteries, while Group 2 included 14 specimens with non-sclerotic arteries. Measurement areas corresponding to the transitional zones between normal and sclerotic tissue were excluded from the analysis because of subsequent results interpretation.

A tube that was 4 mm in diameter, 30 mm in length, and 0.5 mm in thickness was selected for testing as a geometrically suitable phantom for 3D printing of the basilar artery. The diameter and wall thickness were defined according to auxiliary measurements on cadaveric material and correspond to average round values. The measured values are also in accordance with radiological measurements as a representative value for the basilar artery and vertebral artery [19,20]. The phantom samples were printed using six different materials and two 3D-printing techniques in our in-house 3D-printing labs for preoperative brain aneurysm training. All samples were printed in a vertical orientation. The laboratory is certified according to ISO 13485 standards [21]. We have used the following resins for stereolithography (SLA) 3D printing: Flex Gingiva Mask (Prusa, Prague, Czech Republic), Flex Anatomical (Prusa, Prague, Czech Republic), Silicone 40A (Formlabs, Somerville, MA, USA), and Elastic 50A (Formlabs, Somerville, MA, USA). The filaments used for fused-deposition-modelling (FDM) 3D printing were as follows: Fiberflex 40D (Fiberlogy, Brzezie, Poland) and TPE (Latice Service, Loos, France). All printed parts were measured after the fabrication procedure. From each material, five samples were printed, making a total of 30 samples.

**Figure 3:**
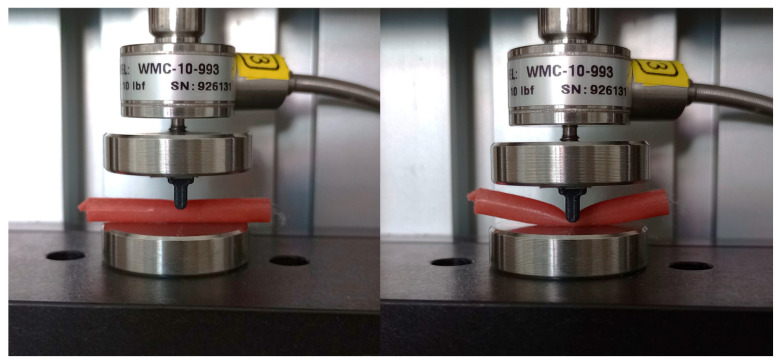
Experimental benchmark mimicking clipping.

The experiments were carried out on a two-column micro-testing machine, eXpert 400, using a 10-pound force gauge (Admet, Norwood, NC, USA). The experiment consisted of mimicking the clipping mechanism of the brain artery on the length of the artery. The samples were loaded via a linear punch with a diameter of 2 mm at a vertical speed of 10 mm/min up to the self-contact of the inner walls of the tube, which corresponds to the diameter of the tube, as shown in Figure 3. Three testing points along the length of the artery were selected. A total of 24 measurements were performed on the cadavers. For the synthetic material, only one testing point in the middle of the tube was used, with 30 measurements in total. The surface of the tube was inspected using a Magus microscope after the experiments (Levehuk, Tampa, FL, USA).

Data were analysed using statistical software SPSS version 21 (IBM Corp., Armok, NY, USA). The statistical significance of the mean difference in those two subgroups was tested with the non-paired Mann–Whitney test, given the low number of that subgroup, and a Pearson’s chi test. Significance level for these tests was set to *p* < 0.05.

## 3. Results

**Figure 4:**
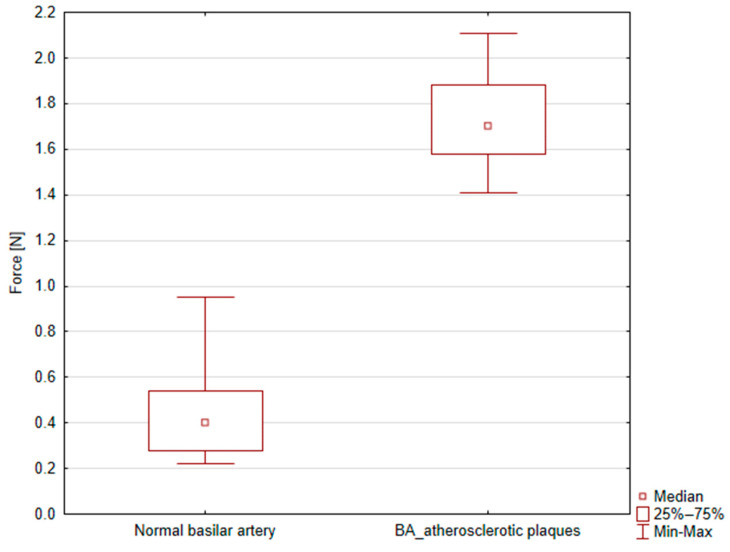
Box plot graph showing peak force [N] for normal and atherosclerotic basilar artery (BA) clipping.

There was a marked difference in mechanical response between regions affected by atherosclerotic plaque and those that were unaffected. The mean maximal force required to fully close the diameter in the unaffected arterial segments was 0.45 ± 0.19 N, whereas the plaque-affected regions required a significantly higher force of 1.73 ± 0.22 N. This difference was statistically significant (*p* = 4.73 × 10^−5^), indicating that atherosclerotic plaques significantly increase the mechanical properties of arterial walls during clipping, as shown in Figure 4.

The force–displacement curves showed shape differences between the two groups. In both cases, the relationship exhibited a highly non-linear character, but the shape and progression of the curves differed notably. In the unaffected arteries, the force increased gradually with displacement, suggesting a more compliant response. In contrast, plaque-affected arteries showed a steeper initial slope and a more abrupt increase in force, reflecting stiffer behaviour likely due to the calcified or fibrotic nature of the plaque.

**Figure 5:**
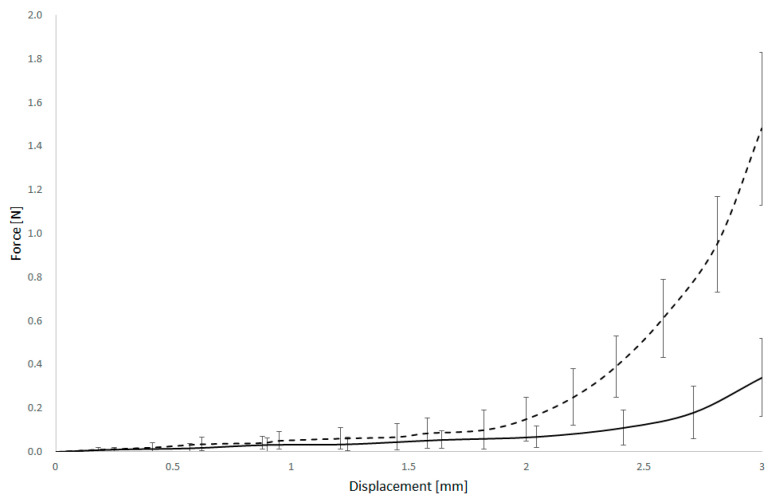
Force and displacement curves of normal (dashed) and arteriosclerotic basilar artery.

Furthermore, the force–displacement profiles often displayed a biphasic character, as shown in Figure 5. The initial phase corresponds to the approach of the arterial walls, with minimal resistance, followed by a second phase where force increases sharply; this inflexion point likely corresponds to self-contact of the deformed arterial walls or the onset of plaque compression.

**Figure 6:**
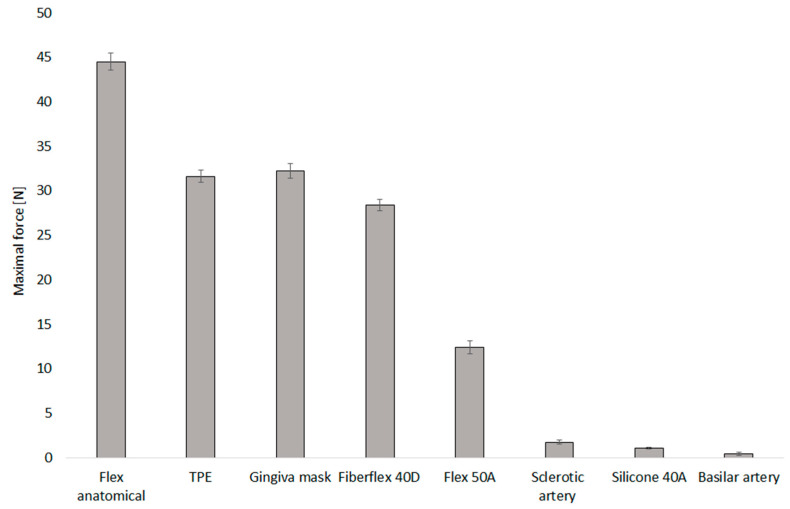
Maximal force for selected 3D-printed materials used for mimicking the brain aneurysm.

The mechanical performance of various 3D-printed arterial models was evaluated to benchmark their suitability for simulating arterial behaviour. The highest mean maximal force was observed in models fabricated with Flex Anatomical material, reaching 44.51 ± 0.98 N, indicating a high degree of stiffness and mechanical resistance. In contrast, models made from Silicone 40A demonstrated the lowest mean maximal force, 1.05 ± 0.12 N, as shown in Figure 6.

Each material exhibited a non-linear force–displacement curve behaviour. Typically, an initial low-slope region was observed, representing the onset of compression and the gradual approximation of internal walls. This was followed by a transition zone characterised by a sharp increase in force, marking the point of wall contact or collapse. The surface inspections after experiments confirmed surface cracks on the Anatomical flex material only; all other materials were without damage after testing.

## 4. Discussion

Mechanical testing of resected aneurysm specimens has shown reduced tensile strength and lower failure strains compared with healthy vessels [22,23,24,25,26]. These properties vary regionally: the aneurysm neck tends to be thicker and stiffer, while the sac and its bulges are thinner, softer, and mechanically more fragile. Finite element modelling studies confirm that these weak zones experience high wall tension and localised stress concentrations, which are closely linked to rupture risk [27,28,29]. From a surgical perspective, clipping introduces further mechanical stresses at the aneurysm neck and adjacent wall; if applied to regions of thin or degenerated tissue, clips may precipitate rupture. Taken together, experimental and computational findings emphasise that aneurysm walls are critically sensitive to external loads, underscoring the need for careful surgical planning and biomechanical insight during clipping procedures.

Aneurysm clips are designed to exert a defined closing force that ensures secure occlusion of the aneurysm neck while minimising damage to the parent vessel or surrounding tissue. Measurements have shown that permanent clips, such as Yasargil or Sugita designs, typically generate closing forces in the range of 1.2–2.0 N, whereas temporary clips exert lower forces, around 0.7–1.1 N, to reduce the vessel wall injury during short occlusions [30,31,32,33,34]. The effective force is not uniform along the blade: it is usually lower at the tip and increases toward the base, reflecting the spring mechanics of the clip. Factors such as blade length, clip geometry, and the material used also influence the applied force. From a biomechanical perspective, an adequate but not excessive closing force is critical, since insufficient pressure risks incomplete occlusion or clip slippage, whereas excessive pressure can crush or tear fragile aneurysm walls.

Our results clearly show that atherosclerotic plaques significantly increase the stiffness of arterial walls, requiring much higher force to close affected regions compared to healthy segments. The force–displacement curves reveal that plaque-affected arteries behave more stiffly, likely due to calcification and fibrosis, which impacts their mechanical response during clipping. When comparing these biological responses to 3D-printed arterial models, none of the tested materials closely matched the mechanical behaviour of real arteries. While materials like Flex Anatomical showed high stiffness and Silicone 40A was more compliant, they all differed substantially in force magnitudes and deformation patterns from biological tissues. This highlights the current limitations of 3D-printed models for accurately replicating the mechanical properties of arteries, especially diseased ones, which is critical for realistic surgical simulation and device testing. Thus, simple anatomical reconstructions based on radiological data are, in our opinion, no longer sustainable. The future consists of the development of 3D-printed materials that closely mimic biological tissue, enabling truly realistic training. Standard uniaxial or similar tests are insufficient to fully capture these mechanics.

Some limitations of our study must be acknowledged. First, the mechanical stiffness of the basilar artery might differ from that of arteries at more typical intracranial aneurysm sites, such as those in the anterior circulation. This difference may influence both the haemodynamic environment and the mechanical interaction with endovascular devices. Additionally, the anatomical configuration of the basilar artery, including its branching pattern and surrounding structures, deviates from more common aneurysm locations. These anatomical variations may limit the generalisability of our findings to aneurysms located elsewhere in the cerebrovascular system. A potential weakness of this study may be the degradation of soft tissues that occurs between sampling and testing. The repeatability of 3D-printed parts also depends on the technology used. SLA produces more precise models, whereas FDM parts show greater variability in printed diameter. The authors of the study tried to minimise this time interval as much as possible; however, they are aware that soft tissues degrade rapidly even in physiological solution. On the other hand, all samples were stored and tested in the same way, so the impact on the variability of the results is unequivocal.

## 5. Conclusions

In the future, we will undoubtedly move towards increasingly realistic models of brain arteries and aneurysms based on 3D-printing technology. This is the best way we can practise real clipping of specific aneurysms before the actual surgery. However, in order to determine the optimal printable material, a series of comparative studies between printable masses and real blood vessel tissue will be necessary. Our study should contribute to the development of further research in this area.

The study also shows that it is necessary to take into account possible sclerotic changes in the vascular wall, which change the nature of the tissue intended for clipping. Atherosclerotic plaques significantly increase arterial stiffness, requiring much higher force to close the plaque-affected segments compared to unaffected arteries. The force–displacement curves show a stiffer, less compliant behaviour in plaque regions, likely due to calcification or fibrosis. Mechanical testing of 3D-printed models revealed varied stiffness, with some materials mimicking native artery behaviour better than others. These results highlight the need to consider plaque effects and material properties when developing realistic vascular models for simulation and testing.

## Data Availability

The original contributions presented in this study are included in the article. Further inquiries can be directed to the corresponding author.
